# Stereoscopic X-ray image and thermo-optical surface guidance for breast cancer radiotherapy in deep inspiration breath-hold

**DOI:** 10.1007/s00066-023-02153-y

**Published:** 2023-10-05

**Authors:** Martin Buschmann, Daniela Kauer-Dorner, Stefan Konrad, Dietmar Georg, Joachim Widder, Barbara Knäusl

**Affiliations:** Department of Radiation Oncology, Comprehensive Cancer Center, Medical University of Vienna/AKH Wien, Währinger Gürtel 18–20, Vienna, 1090 Austria

**Keywords:** Breast cancer, Image-guided radiotherapy, Surface-guided radiotherapy, Motion management

## Abstract

**Purpose:**

To investigate the feasibility of a thermo-optical surface imaging (SGRT) system combined with room-based stereoscopic X‑ray image guidance (IGRT) in a dedicated breast deep inspiration breath-hold (DIBH) irradiation workflow. In this context, benchmarking of portal imaging (EPID) and cone-beam CT (CBCT) against stereoscopic X‑rays was performed.

**Methods:**

SGRT + IGRT data of 30 left-sided DIBH breast patients (1 patient with bilateral cancer) treated in 351 fractions using thermo-optical surface imaging and X-ray IGRT were retrospectively analysed. Patients were prepositioned based on a free-breathing surface reference derived from a CT scan. Once the DIBH was reached using visual feedback, two stereoscopic X‑ray images were acquired and registered to the digitally reconstructed radiographs derived from the DIBH CT. Based on this registration, a couch correction was performed. Positioning and monitoring by surface and X-ray imaging were verified by protocol-based EPID or CBCT imaging at selected fractions and the calculation of residual geometric deviations.

**Results:**

The median X‑ray-derived couch correction vector was 4.9 (interquartile range [IQR] 3.3–7.1) mm long. Verification imaging was performed for 134 fractions (216 RT field verifications) with EPID and for 37 fractions with CBCT, respectively. The median 2D/3D deviation vector length over all verification images was 2.5 (IQR 1.6–3.9) mm/3.4 (IQR 2.2–4.8) mm for EPID/CBCT, both being well within the planning target volume (PTV) margins (7 mm). A moderate correlation (0.49–0.65) was observed between the surface signal and X-ray position in DIBH.

**Conclusion:**

DIBH treatments using thermo-optical SGRT and X-ray IGRT were feasible for breast cancer patients. Stereoscopic X‑ray positioning was successfully verified by standard IGRT techniques.

## Introduction

Over the last decades, breast-conserving surgery followed by radiotherapy was established as the standard of care treatment for early stage breast cancer [1]. However, the exposure of the heart and lungs to ionizing radiation during treatment increases the risk of heart disease and secondary lung cancer [2, 3]. To reduce the risk of radiation-related side effects from external beam therapy, the technique of deep inspiration breath hold (DIBH) was established [4, 5].

During DIBH the patient holds air in their lungs at a predefined lung volume close from the end of inspiration for the time of the irradiation to increase the distance between the heart and the treatment area to reduce the exposure of the heart and lungs. It can further stabilise the patients’ position.

DIBH is especially promising for left-sided breast cancer patients receiving whole or partial breast irradiation, where the heart is close to the irradiated volume. Several in silico studies demonstrated reduced organs-at-risk (OAR) doses and potential risks of side effects [6, 7]. Since DIBH is a relatively new treatment technique a reduction of long-term side effects in a clinical trial has not been reported yet but might be expected in the near future as DIBH becomes the new clinical standard. However, Mast et al. [8] already demonstrated that DIBH leads to reduced coronary artery calcium scores.

The DIBH manoeuvre requires accurate breathing monitoring to guarantee reproducible patient positioning during irradiation. One of the most sophisticated technologies for patient monitoring is optical surface imaging, which enables continuous nonradiographic and noninvasive assessment of a patient’s surface [9, 10]. Surface-guided radiotherapy (SGRT) should, however, be combined with internal imaging because the correlation of surface motion with target motion is not guaranteed [11]. Verification of surface-guided breast radiotherapy is commonly performed by portal imaging [12, 13], cone-beam CT (CBCT) [14] or kV–kV pairs [15]. The high accuracy of CBCT becomes especially important for rotational delivery techniques employing nonstatic fields, like volumetric modulated arc therapy (VMAT). Recently, a system combining thermo-optical surface imaging with room-based stereoscopic X‑ray image-guided radiotherapy (IGRT) was commercially released (Exactrac Dynamic v1.1 (ETD), Brainlab AG, Munich, Germany) with a dedicated DIBH workflow [16]. Stereoscopic room-based X‑ray imaging is an established technique for cranial treatment and positioning of pelvic sites [17, 18], but the use for breast cancer irradiation, especially with DIBH, has so far not been described in the literature. Stereoscopic X‑ray imaging has the potential to enable faster treatments (no gantry rotations necessary) and/or reduce the imaging dose burden compared to portal imaging or CBCT [19]. Another novelty of the ETD system is the use of thermo-optical surface imaging, whereas established systems use optical wavelengths only [15]. SGRT with this extended wavelength range including infrared is still unexplored in the scientific literature although it is rapidly being adopted in clinical treatments.

This study aims to retrospectively evaluate the combination of those novel technologies, stereoscopic IGRT and thermo-optical SGRT, in the dedicated DIBH workflow and to compare with established IGRT techniques.

## Materials and methods

### Patient data, imaging and treatment

Thirty patients (average age 56 years, range 40–78 years) treated postoperatively for left-sided breast cancer were included in this retrospective analysis. These were the first patients treated with the novel ETD DIBH workflow for at least fractions. This retrospective study was registered with the institutional ethical review board (study number: 1248/2023). The prescription was 40.05 Gy in 15 fractions to the whole breast or the tumour bed as partial breast irradiation. For 20 whole breast irradiations, a subsequent boost to the tumour bed was planned with 10 Gy in 4 fractions. One patient received bilateral breast radiotherapy in DIBH counting as two separate treatments. The clinical target volume (CTV) for partial breast irradiation was derived by adding an 8 mm margin around the delineated tumour bed. The planning target volume (PTV) margin to account for setup uncertainties was 7 mm. Target volume details are summarized in Table 1.

**Table 1:** Target volumes and treatment techniques of the patient cohort

*Treatment concept*	*WBI*	*PBI*	–
Patients, *n*	27	3	–
CTV range, cm^3^	136–1359	8–73	
*Treatment techniques*	*3DCRT*	*IMRT*	*VMAT*
Patients, *n*	3	23	4
Patients with boost, *n*	10	10	0
Beam modulation	Wedge	Up to 4 segments	1–2 arcs
Average (range) of MUs	390 (210–713)	260 (204–588)	524 (417–600)

*WBI* whole breast irradiation, *PBI* partial breast irradiation, *3DCRT* 3D conformal radiotherapy, *IMRT* intensity modulated radiotherapy, *VMAT* volumetric modulated arc therapy, *MU* monitor unit, *CTV* clinical target volume

A total of 351 fractions were analysed, as not all included patients received their full treatment course following the ETD workflow (described in the Independent in-room image verification section), due to service slot interruptions. A total of 374 ETD positioning datasets were analysed, for those 351 fractions as repositioning (including repeated IGRT and couch correction) was necessary for 7% and one ETD dataset could not be retrieved.

All patients were immobilized with a breast board (BreastSTEP, IT‑V, Innsbruck, Austria) during imaging and irradiation. For treatment planning computed tomography (CT) scans in 2 mm slice thickness were acquired on Siemens Somatom Definition AS CT scanner (Siemens Healthineers AG, Erlangen, Germany) in two breathing states: DIBH and free-breathing (FB). The inspiration breath-hold level was set by guiding the patient through several breath-holds and establishing a reproducible and stable inspiration amplitude. Visual guidance was provided to the patient by using the Sentinel surface scanner (C-Rad, Uppsala, Sweden) and a head-mounted tablet in the CT room. A low-dose CT protocol was introduced for the FB-CT that reduced the imaging dose by 45% compared to the DIBH-CT.

The two CT scans were imported into the RayStation treatment planning system (TPS) v11a (RaySearch Laboratories, Stockholm, Sweden) in the same frame-of-reference if possible. In case the patient moved between scans a spine registration had to be performed in the TPS. All targets, OARs and the body contour were delineated on the DIBH-CT scan for treatment plan optimization. On the FB-CT only the body contour was delineated and transferred to the DIBH-CT. The two body contours were stored in the ETD system to derive the inspiration amplitude from the distance of the contours in ventral direction when setting the DIBH-POI at the first fraction (see “ETD DIBH workflow” section).

All treatments used 6 or 10 MV beams and the dose was calculated with the collapsed cone dose calculation engine (v5.5) of the TPS. Details of the treatment techniques are summarized in Table 1.

Patients were treated at two identical ELEKTA Agility linacs (Elekta AB, Stockholm, Sweden) equipped with a Hexapod couch with 6 degree of freedom (DOF). In addition, the linacs were equipped with a standard electronic portal imaging device (EPID) iView system (Elekta AB, Stockholm, Sweden) and cone-beam CT (CBCT) XVI system (Elekta AB, Stockholm, Sweden).

The ETD v1.1 system (Brainlab AG, Munich, Germany) was used for DIBH monitoring. The ETD system consists of a room-based stereoscopic radiographic system [20] and a ceiling-mounted thermo-optical scanner (OS). Two oblique X‑ray imagers provide a field size of 18 × 18 cm^2^ at the isocentre. Radiation qualities of 40 to 150 kV are available.

The combined OS-thermal camera is positioned on the ceiling in the centre where the field-of-view (FOV) is not blocked by the linac gantry at any angle. The housing contains two stereoscopic three-dimensional (3D) data cameras positioned at a distance of approx. 20 cm, a thermal camera and a structured light projector. The manufacturer specifies a camera acquisition speed of up to 20 images per second and a scanning volume of 64 × 49 × 40 cm^3^.

A monitor is mounted on an adjustable arm in the treatment room for visual guidance for the patient, which is visible in combination with a mirror on the head end of the treatment couch. A more extensive description of the novel ETD system can be found in the literature [16, 21].

### ETD DIBH workflow

**Fig. 1:**
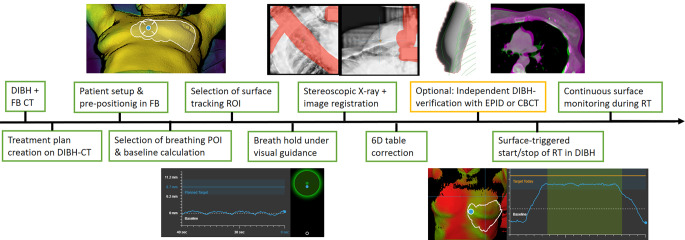
Sketch of the Exactrac Dynamic Deep inspiration breath-hold workflow from CT imaging to treatment monitoring. *CBCT* cone-beam computed tomography, *DIBH* deep inspiration breath hold, *EPID* electronic portal imaging device, *FB* free-breathing, *POI* point-of-interest, *ROI* region-of-interest, *CT* computed tomography

**Fig. 2:**
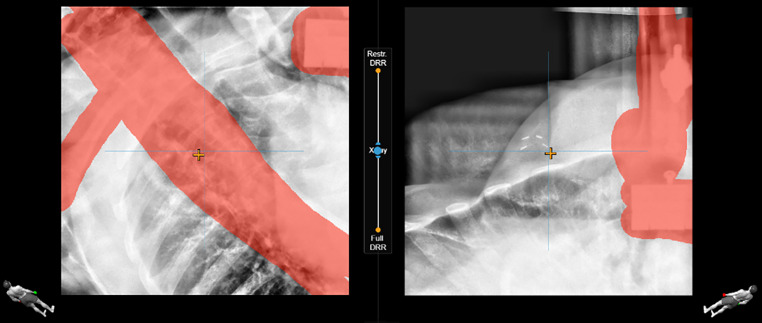
Example of stereoscopic X‑ray image guidance (IGRT) for patient positioning with a focus on the left-sided ribs. *Red* Red areas are neglected in the image registration to achieve a good match of the ribs.

A sketch of the whole workflow is depicted in Fig. 1. During the prepositioning (initial patient setup), the real-time surface image of the patient was registered to the external contour from the FB-CT by the ETD software. This way the patient was moved into the treatment position based on 3D deviation vector information. For monitoring the breathing curve of the patient, a point of interest (POI) was defined at the sternum. For surface surveillance, a region of interest (ROI) including the treated breast was defined in FB. The breathing amplitude at the sternum POI was averaged over 10 s during FB to derive a mean FB baseline level. The planned DIBH amplitude was defined as the vertical distance between the DIBH and FB body contours at the position of this POI. The live breathing amplitude and the planned DIBH level with a gating window of 5 mm (±2.5 mm of the DIBH level) were then projected on the monitor for visual feedback for the patient. The DIBH body contour from the TPS was now activated as a reference surface. As soon as the patient’s respiratory signal reached the gating window, an X‑ray image was acquired (default X‑ray generator settings: 90 kV, 10 mAs) together with a daily reference DIBH surface for monitoring to assure the matching of the X‑ray position and the target surface. The breathing level during X‑ray acquisition was set as the daily reference. The X‑rays were matched to digitally reconstructed radiographs (DRR) from the planning DIBH-CT. Matching was based on bony anatomy and the registration ROI/VOI was manually adjusted to focus the automatic registration on the relevant region. The spine was generally excluded from the registration ROI and special attention was given to a good match of the left-sided ribs as a surrogate for the left breast (Fig. 2). Based on the registration, 6D correction couch shifts were calculated and then applied with the Hexapod table. If a manual image registration was performed, only a 3D match was reported (all rotations zero) and sent to the treatment couch. Rotations > 3° cannot be performed by the robotic table; therefore a 6D registration compromise was calculated by reducing larger rotations to 2.9°. If a visually acceptable X‑ray match was not achievable with compromised rotations, patient posture correction and prepositioning had to be repeated. Within this study, the registration results of the original 6D match were used for evaluation.

Intrafraction motion monitoring during irradiation was based on the DIBH-surface ROI and the breathing signal DIBH-POI. This surface area was tracked by the OS-thermal camera and rigidly registered in 6 DOF to the daily reference DIBH-surface ROI. As soon as the surface ROI or the gating POI moved outside predefined tolerances during irradiation the beam was stopped. The default values for the tolerance were based on the PTV margin size and previous SGRT experience: 5 mm (±2.5 mm) gating window, 4.5 mm and 2.5° surface tolerance.

In case unacceptable patient motion was detected during the treatment session, the ETD DIBH workflow had to be performed from start and the patient was repositioned before treatment continuation.

The ETD software recorded three 6D vectors for each positioning procedure:

- Remaining surface shift after free-breathing prepositioning,
- Surface shifts at X‑ray acquisition in breath-hold (relative to the DIBH-CT surface), and
- X‑ray-based correction (relative to DIBH DRR) applied to the table.

### Independent in-room image verification

The positioning with the ETD system was verified with independent IGRT techniques based on the respective IGRT protocols. The anatomical view on the oblique radiographs hinders visual interpretation of the calculated shift as it only visualises bony structures but not the soft tissue defining the outer border of the irradiated breast tissue. Therefore, regular verification with EPID or CBCT imaging was performed in a separate breath-hold manoeuvre after stereoscopic X‑ray positioning. This independent verification was performed to ensure safe clinical treatments with a novel workflow that relies on stereoscopic X‑rays as sole IGRT tool for couch corrections.

For 3D conformal radiotherapy (3DCRT) and intensity modulated radiotherapy (IMRT) treatments, portal image verification of the treatment fields (single exposure, largest segment covering the full treatment field for IMRT) was performed for the first fractions and at least weekly thereafter. For partial breast or boost treatments double exposure portal images were acquired at the first fraction. For volumetric modulated arc therapy (VMAT) treatments CBCT verification was performed with the same schedule as the single exposure fields. Occasionally also 3DCRT and IMRT treatments were verified by CBCT due to clinical and technical reasons. During EPID and CBCT registration the focus was on the thoracic wall and breast contour. Calculated shifts from the verification IGRT acquisitions could not be applied to the treatment table in the ETD DIBH workflow for technical reasons. As EPID and CBCT are still considered the IGRT gold standard, the shifts derived from EPID or CBCT in a subsequent breath hold after ETD-based positioning were recorded as residual positioning errors. For EPID or CBCT images, a 2D or 3D (3 translations + optional 3 rotations) deviation vector was derived, respectively. After ETD positioning, verification imaging was performed for 134 fractions (216 RT field verifications) with EPID and for 37 fractions with CBCT, respectively. In case of large positional discrepancies in the EPID or CBCT images, the ETD DIBH workflow was repeated including couch corrections until verification was successful.

### Evaluation

Deviations between the different image modalities were analyzed over all included fractions and median values and interquartile ranges (IQR, 25th and 75th percentiles of the data) were calculated. To investigate the agreement of SGRT and stereoscopic IGRT, the surface-derived coordinates in FB and DIBH were correlated with the X‑ray-based vectors using the Pearson correlation coefficient. The two-sided significance level for rejecting the null hypothesis that the distributions underlying the samples are uncorrelated was set to *p* < 0.01.

## Results

**Fig. 3:**
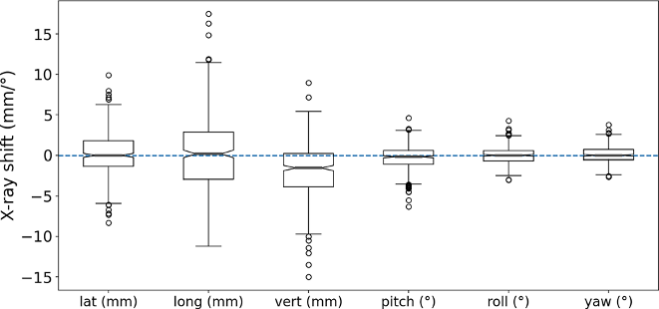
The 6D registration result of the stereoscopic X‑ray in DIBH and shift applied to the table. The *box* extends from the first quantile to the third quantile and the *line in the box* represents the median. The *whiskers* indicate the highest/lowest value below/above: first/third quartile ±1.5 × interquartile range. The *circles* represent outliers. *lat* lateral, *long* longitudinal, *vert* vertical

Considerable ETD X‑ray-derived shifts had to be performed after prepositioning in FB. The median X‑ray-based correction 3D shift vector in DIBH was 4.9 (IQR 3.3–7.1) mm long (lateral: 0.0 [IQR −1.3 to 1.8] mm, longitudinal: 0.2 [IQR −2.9 to 2.8] mm, vertical: −1.5 [IQR −3.9 to 0.2] mm, pitch: −0.2 [IQR −1.1 to 0.6] °, roll: 0.0 [IQR −0.7 to 0.6] °, yaw: 0.0 [IQR −0.5 to 0.7] °, Fig. 3).

**Fig. 4:**
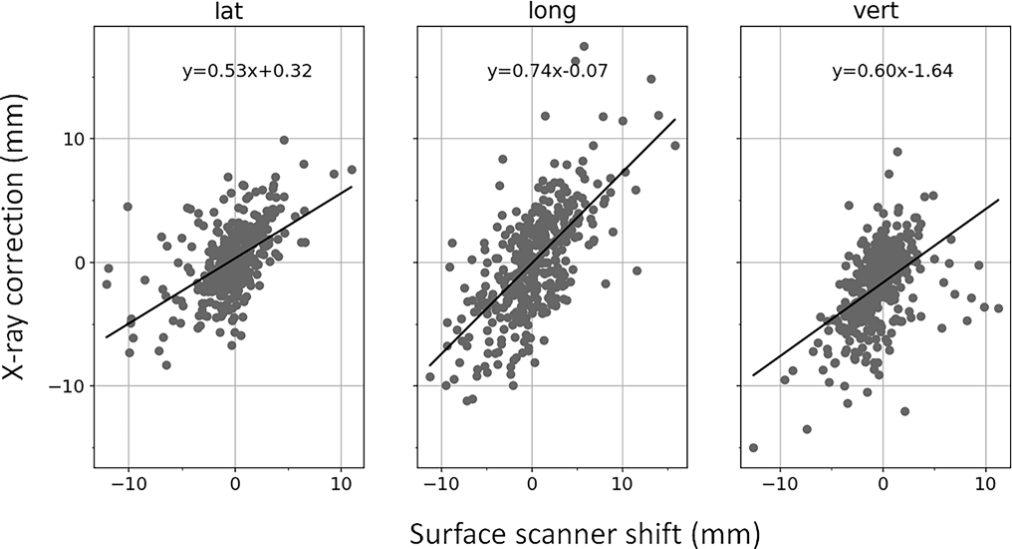
Correlation of surface shift vs. X‑ray correction, both acquired at the same moment in deep inspiration breath-hold (DIBH). *Black lines* represent the linear fit to the data. Pearson correlation coefficient: lateral [lat] 0.54, longitudinal [long] 0.65, vertical [vert] 0.49.

A moderate and statistically significant correlation (R = 0.49–0.65) between the DIBH surface signal and DIBH X‑ray position was observed. In Fig. 4 the surface shifts at the moment of X‑ray acquisition in breath-hold are plotted against the X‑ray-based table shifts.

**Fig. 5:**
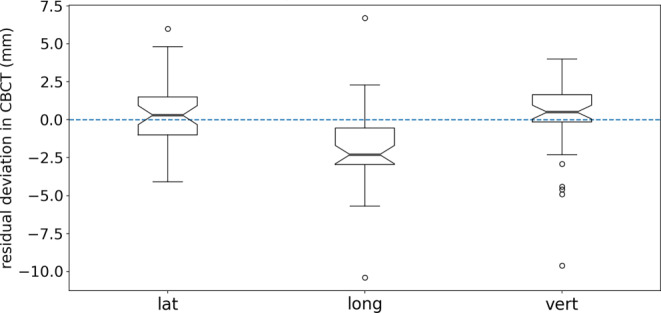
Boxplot of residual deviations present in cone-beam computed tomography (CBCT) analysis after patient positioning based on stereoscopic X‑rays in 37 fractions. The largest deviations were seen for fractions for which repositioning was performed. The *box* extends from the first quantile to the third quantile and the *line in the box* represents the median. *Whiskers* indicate the highest/lowest value below/above: first/third quartile ±1.5 × interquartile range. *Circles* represent outliers.* lat* lateral, *long* longitudinal, *vert* vertical.

The median 2D/3D deviation vector length over all verification images was 2.5 (IQR 1.6–3.9) mm/3.4 (IQR 2.2–4.8) mm for EPID/CBCT. Both measures were well within the PTV margins (7 mm), which demonstrates accurate X‑ray based positioning. The CBCT verification results showed a systematic residual component in longitudinal direction (Fig. 5). This systematic deviation could not be confirmed in the vertical shift in the EPID verification images (median −0.5 [IQR −2.0 to 1.2] mm) which is also oriented in longitudinal direction irrespective of the gantry angle.

In 24 fractions (7%), repositioning with ETD was performed during the treatment session (before or after start of irradiation). During seven out of these 24 repositioning manoeuvres (repeated) EPID or CBCT verification images were acquired.

For 4 of 24 fractions, repositioning before irradiation was performed after a disagreement (> 4.8 mm in at least one direction) between ETD imaging and verification imaging. In those cases, repositioning with the ETD workflow improved the agreement.

## Discussion

The described workflow efficiently combines SGRT and IGRT and is in line with recent SGRT and DIBH guidelines [10, 11, 22] since the correlation of the surface signal to internal anatomy cannot be reliably predicted by SGRT alone. Positioning was performed based on IGRT and subsequent intrafraction monitoring and breathing guidance was provided by SGRT. The performance of the stereoscopic X‑ray system for DIBH positioning was benchmarked against established IGRT techniques, i.e. portal imaging and CBCT (Fig. 5). The results demonstrated sufficient geometrical agreement between these IGRT modalities and the room-based X‑ray system may be used as the sole IGRT system for daily breast DIBH positioning in the future. However, weekly verification with a second IGRT method is recommended due to the difficulty to interpret image geometry in the oblique radiographs and the limited soft breast tissue visualization on the X‑rays. The cause of residual deviations—systematic differences between the imaging modalities or the limited reproducibility of the DIBH manoeuvre—remains subject to further investigations. Detected disagreement between X‑ray and verification images could either be caused by patient movement or pronounced breathing differences between the two image acquisitions or due to image-modality-inherent characteristics or issues in system handling [23]. EPID and X-ray images are acquired in an instant, whereas the acquisition time of a breast CBCT in DIBH is approximately 40 s. During this time a potential anatomical shift below the SGRT thresholds may occur as a patient slowly releases air from her lungs [23]. Those circumstances might explain the difference in longitudinal registration results between CBCT and EPID verification. However, the results are reassuring that our institutional breast margin of 7 mm is an appropriate choice. Residual rotations in the CBCT verification were not reported as many CBCT registrations were performed manually in 3D for simpler and more efficient manual matching. For the registrations where rotations were included the maximum residual rotational deviation was 3.4° roll.

Using stereoscopic X‑rays for patient positioning can result in a considerable reduction of treatment session time because the gantry with the imager does not need to be rotated around the patient for orthogonal MV, onboard kV-kV or CBCT imaging. In addition, the imaging dose can be substantially reduced compared to (double exposure) EPID imaging and CBCT scans [19]. Imaging doses were not measured in our study; however, based on the ETD acceptance measurements a dose–area product of approx. 10 µGym^2^ per fraction was extrapolated.

Da Silva Mendes et al. demonstrated an agreement better than 0.5 mm between the thermo-optical surface position and the X‑ray position of static phantoms using the ETD system and reported on the first cranial patient treatments [16]. Our study represents the first application of thermo-optical surface-guided radiotherapy in breast radiotherapy with DIBH. In contrast to many other SGRT systems, the ETD system uses only one camera unit instead of three in the treatment room. The additional infrared signal component should facilitate accurate surface tracking with just one camera system. However, the shading of the surface FOV by the patient itself needs to be considered and may be a disadvantage compared to a three-camera setup [10]. Low breast board inclination, obesity and larger breast size were unfavourable factors for accurate surface monitoring in this study and the board inclination was therefore increased from 0 to 15° after the first 5 patients. A direct comparison between the performance of optical and thermo-optical tracking was outside the scope of this work, but should be investigated in future studies.

To our knowledge, this is the first report using stereoscopic X‑ray guidance for patient positioning in breast DIBH radiotherapy. Ciérvide et al. [24] investigated stereoscopic kV-IGRT for accelerated partial breast irradiation in free breathing and reported geometric uncertainties below 5 mm. Our study demonstrated that such a DIBH workflow is feasible for breast cancer treatments.

Lorchel et al. reported reproducibility of breath holds within 3 mm when using a surface scanner [25]. The DIBH reproducibility may be increased by decreasing the gating window for visual guidance. A value of 5 mm was chosen for the included patient cohort, but smaller values of 3–4 mm were previously reported [26, 27]. A smaller gating window may make it more difficult to hold their breath within the window for some patients. In a small percentage of fractions, repositioning with the ETD workflow had to be performed. The reason for repositioning cannot be determined due to the retrospective nature of this study but might be explained by patient movement after the first positioning procedure, software crashes or unsuccessful verification with EPID or CBCT.

The data evaluated in this manuscript underlined that patient positioning based only on surface image information in FB may not provide sufficient anatomic information since substantial residual shifts were detected with stereoscopic IGRT after FB SGRT-based positioning (Fig. 3). The correlation results showed that surface-based positioning in FB was weakly correlated to the stereoscopic X‑ray localization in DIBH (R = 0.30–0.35, data not shown in detail). Furthermore, the surface shift in DIBH was only moderately correlated with the DIBH X‑ray shift (Fig. 4), even these two shifts were recorded during the same DIBH manoeuvre and breath hold variations were thereby eliminated. However, the correlation of surface signal with internal motion may strongly depend on the choice of surface ROI [14]. Those results strongly confirm the recommendation for caution with surface-only workflows and the use of daily breathing baseline correction.

## Conclusion

Deep inspiration breath-hold treatments for breast radiotherapy with stereoscopic X‑ray image-guided radiotherapy (IGRT) and thermo-optical surface-guided radiotherapy were feasible. Exactrac Dynamic X‑ray positioning was successfully verified by established IGRT techniques.
